# The role of postmastectomy radiation in patients with ypN0 breast cancer after neoadjuvant chemotherapy: a meta-analysis

**DOI:** 10.1186/s12885-021-08423-1

**Published:** 2021-06-25

**Authors:** Ke Wang, Xiaoyan Jin, Weilan Wang, Xiuyan Yu, Jian Huang

**Affiliations:** 1 Department of Breast Surgery, Second Affiliated Hospital of Zhejiang University School of Medicine, 88 Jiefang Road, Hangzhou, 310009 China; 2 Department of Surgical Oncology, Taizhou Municipal Hospital, Taizhou, 318000 Zhejiang Province China; 3 Department of Breast Surgery, Changxing Hospital, Second Affiliated Hospital of Zhejiang University School of Medicine, Huzhou, 313100 China

**Keywords:** Breast cancer, Neoadjuvant chemotherapy, Postmastectomy radiation therapy, Negative lymph nodes, Prognosis, Meta-analysis

## Abstract

**Background:**

It has been demonstrated that postmastectomy radiation therapy (PMRT) was beneficial for breast cancer patients who are axillary lymph node-positive. However, the effectiveness of radiotherapy in pathological negative nodes (ypN0) after neoadjuvant chemotherapy (NAC) remains open to considerable debate. Here, we aim to evaluate whether PMRT improves loco-regional control and survival for such patients.

**Methods:**

The literature from January 2004 to June 2019 was searched. The effects of PMRT on local-regional recurrence (LRR) and survival was evaluated in a meta-analysis. Pooled relative risk (RR) values with 95% confidence intervals (CIs) were computed using random and fixed-effect model. Subgroup and heterogeneity analyses were also conducted.

**Results:**

Twelve studies that included 17,747 patients met the inclusion criteria. Pooled results showed that PMRT was associated with reduced LRR (RR, 0.38; 95% CI, 0.19–0.77, *P* = 0.007), particularly in patients with stage III breast cancer (RR, 0.16; 95% CI, 0.07–0.37, *P* < 0.001). However, no significant difference in disease-free survival were observed with the addition of PMRT for ypN0 patients (RR, 0.70; 95% CI, 0.21–2.27, *P* = 0.55). Also, there was no statistically significant association between radiotherapy with overall survival (RR, 0.81; 95% CI, 0.64–1.04, *P* = 0.10).

**Conclusions:**

Our meta-analysis indicated that PMRT might reduce local-regional recurrence for ypN0 patients after NAC, but lack of benefit for survival outcomes. Prospective randomized clinical trial data will be needed to confirm our results.

**Supplementary Information:**

The online version contains supplementary material available at 10.1186/s12885-021-08423-1.

## Background

Neoadjuvant chemotherapy (NAC) is increasingly used in locally advanced breast cancer. The advantages of NAC include that it can reduce pathologic stage and increased potential breast conservation therapy, as well as upfront treatment of micrometastatic cancer [1, 2]. In the adjuvant setting, postmastectomy radiation therapy (PMRT) have shown significantly reduces recurrence in patients with positive lymph nodes after following systemic treatment [3–5]. However, the discrepancy in the response to NAC complicates the adjuvant setting treatment decision, and therefore, the role of PMRT in breast cancer with no evidence of residual pathologic nodal disease (ypN0) has remained controversial.

Recently, several retrospective studies found that PMRT was associated with improving both local control and survival in ypN0 breast cancer patients [6–8]. A recent analysis reported that PMRT reduced local-regional recurrence (LRR) in ypN0 breast cancer cases, following primary systemic treatment [9]. Subgroup analysis further confirmed a significant survival benefit in patients with cT1-2 N1 breast cancer. However, other retrospective data showed that patients with pathologically node-negative (ypN0) disease after NAC had smaller absolute rates of 5-year local-regional recurrence (LRR) (8% in the PMRT group vs. 12% in the non-PMRT group) [10]. The results implied that there was no increase in the risk of LRR when PMRT was omitted.

The aim of gaining better insight into the effectiveness of PMRT in patients after NAC, we carried out the first comprehensive meta-analysis and systemic review on this topic, particularly those studies that evaluated patients presenting with clinically negative lymph nodes who achieved a favorable pathologic response to NAC. Then, we investigated the prognostic value of PMRT (presence vs. absence) on LRR and survival (DFS or OS), as well as performing subgroup analyses.

## Methods and materials

### Literature search

In this systematic review and meta-analysis, literature in the PubMed database and major oncology congress abstracts were searched in June 2019, and the following search terms were variably combined: “breast cancer,” “breast carcinoma,” “breast neoplasm,” “neoadjuvant chemotherapy,” “preoperative chemotherapy,” “radiation therapy,” “postmastectomy radiotherapy”. As some trials concerning NAC and breast cancer prognosis may not have yet been published, we searched for relevant abstracts published in major international proceedings. We also reviewed references of relevant published trials to identify additional study articles.

### Eligibility criteria

All studies that met the following criteria were included: patients diagnosed with breast cancer, achievement of a pathological negative node response to NAC, adequate data provided for estimating the relative risk (RR) for LRR and survival outcome, and original articles. Histological type and breast cancer status were not restricted. Exclusion criteria were as follows: studies published in a systematic review, commentaries, letters, or the study did not determine LRR or survival.

### Data extraction

Two investigators independently extracted data from potentially eligible studies. Any disagreement was resolved by discussion. The data were collected directly from the publication: first author’s name, country, publication year, number of patients, pathological stage, neoadjuvant chemotherapy, type of surgery, outcome and median follow-up duration. If LRR or survival information was not direct provided, we calculated the data from the available Kaplan–Meier curves to estimate the clinical outcomes [11]. The relevant information was carefully extracted according to the following three aspects: (1) PMRT was correlated with LRR in breast cancer and subgroup; (2) PMRT was associated with DFS or OS; (3) the ypN0 (include pCR) was used as eligible definition.

Pooled relative risk (RR) values with 95% confidence intervals (CIs) were calculated by random or fixed-effect model. The test for heterogeneity was using Cochran’s Q method and the I^2^ statistic. When the *P*-value was more than 0.05, a fixed-effect model estimate was presented. Otherwise, the random-effects estimate was considered. Potential publication biases were assessed with the funnel plot, as well as the Egger and begg tests. Sensitivity analyses were conducted to estimate the influence of individual studies from the meta-analysis. All statistical tests were conducted using R-based open-source meta-analysis software (version 2.14.2).

## Results

### Description of the studies

**Fig. 1:**
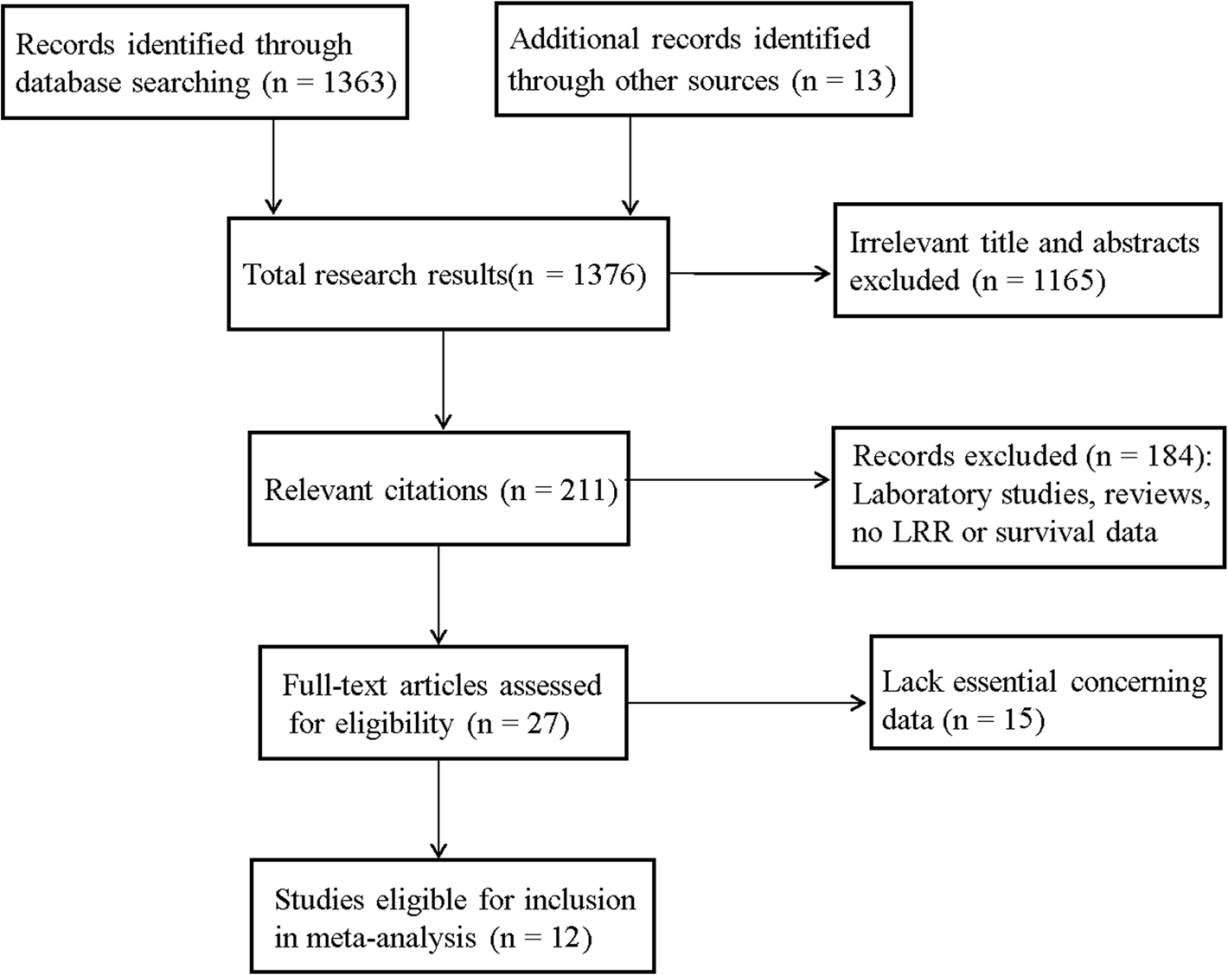
Study flow chart

After searching the databases, 1376 records were fully reviewed (Fig. 1). However, 1165 were excluded after screening of titles and abstracts. Another 184 studies were excluded for the following reasons: they were laboratory studies or irrelevant studies, study sample sizes were too small (size ≤30), and they were duplicate articles or provided no LRR or survival outcome data. Another 15 articles were further excluded because essential results could not be extracted from the data. Finally, 12 studies met the criteria for evaluation, comprising 17,747 cases [9, 10, 12–21]. Cohorts ranged from 88 to 8321 patients, and studies were published between 2004 and 2019. The main features of the eligible studies included are summarized in Table 1.

**Table 1:** Selected characteristics of studies in the meta-analysis

Study	Country	Year	No. of patients	Patholo-gical stage	Type of surgery	NAC	Outcome	Median follow up (Months)
Huang	American	2004	676	II -IV	Mastectomy	A/T,VACP	LRR,	69
McGuire	American	2007	106	I-III	Mastectomy	A/T	LRR, OS	62
Scodan	France	2010	134	II-III	Mastectomy	A/T	LRR, DFS, OS	91
Nagar	American	2011	162	II	Mastectomy	A/T	LRR	75
Bae	Korean	2012	98	0- III	Mastectomy	NR	LRR, DFS, OS	42
Shim	Korea	2013	151	II-III	Mastectomy	A/T	LRR, OS, DFS	59
Liu	American	2015	1560	II -III	Mastectomy	NR	OS	56
Rusthove-n	American	2016	3040	II -III	Mastectomy	NR	OS	41
Kantor	American	2016	8321	I-III	Mastectomy	NR	OS	69
Qinlin	China	2017	185	II - III	Mastectomy	A/T	LRR, DFS, OS	70
Cao	China	2017	88	I-III	Mastectomy	A/T	LRR, DFS, OS	67
Miyashita	Japan	2019	3226	I-III	Mastectomy	NR	LRR, DDFS, OS	> 60

*Abbreviations*: *NAC* Neoadjuvant chemotherapy, *A* Anthracycline-based chemotherapy, *A/T* Anthracycline- or taxane- based chemotherapy, *NR* No report, *LRR* Local-regional recurrence, *DFS* Ddisease-free survival, *OS* Overall survival

### Impact of PMRT on LRR in patients with ypN0 breast cancer

**Fig. 2:**
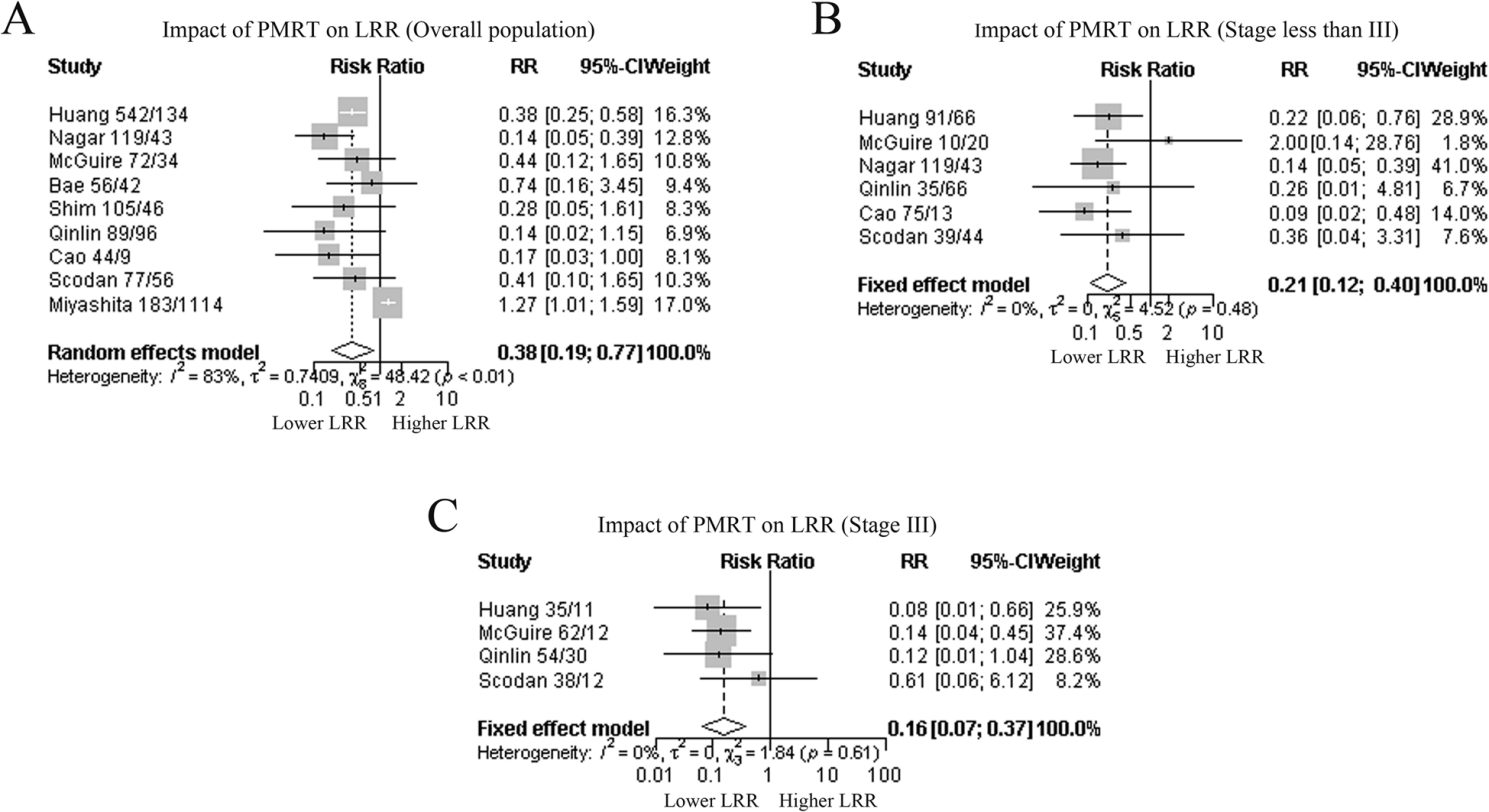
Forrest plot of RR used to evaluate the association between PMRT and LRR. **A** Overall population; **B** Stage less than III breast cancer; **C** Stage III breast cancer. RR values with 95% confidence intervals for survival are associated with PMRT group versus no PMRT group; RR less than 1 represents a lower risk of recurrence. The pooled results showed that PMRT was associated with reduced LRR. *Abbreviations*: PMRT, postmastectomy radiation therapy; LRR, local-regional recurrence

Data for the effect of PMRT on LRR were available from nine studies, accounting for 2861 patients. We evaluated the association between PMRT and LRR in the entire group and in subgroups to determine their contribution. Among those patients with ypN0 breast cancer, the estimated pooled RR for all studies showed that PMRT significantly reduced LRR in patients compared with those in the non-PMRT group (RR, 0.38; 95% CI, 0.19–0.77, *P* = 0.007; Fig. 2A). As the heterogeneity among studies was significant (I^2^ = 83.5%, *P* < 0.001), the random-effects model was applied. The funnel plot and Egger’s test indicated that slight asymmetry in the presence of publication bias of the included literature (Supplementary Figure S1 and Supplementary Table S1). One-way sensitivity analysis confirmed the stability across the included studies.

Subgroup analyses were performed for LRR on the effect of PMRT in various tumor stages. Since heterogeneity across studies was not significant (I^2^ = 0.0%, *P* = 0.48), the fixed-effect model was applied. We noted that PMRT was associated with a lower LRR rate (RR, 0.21; 95% CI, 0.12–0.40, *P* < 0.001) in patients with less than stage III disease (Fig. 2B). Among those with stage III breast cancer, the LRR rate was also significantly reduced for breast cancer patients in the PMRT group compared with those in the non-PMRT group (RR, 0.16; 95% CI, 0.07–0.37, *P* < 0.001; Fig. 2C), which the heterogeneity among studies was not significant (I^2^ = 0.0%, *P* = 0.61). We also calculated random-effects model for subgroup analyses, which the same conclusion was reached (Supplementary Figure S1).

### Effects of PMRT on disease free survival (DFS) in patients with ypN0 breast cancer

**Fig. 3:**
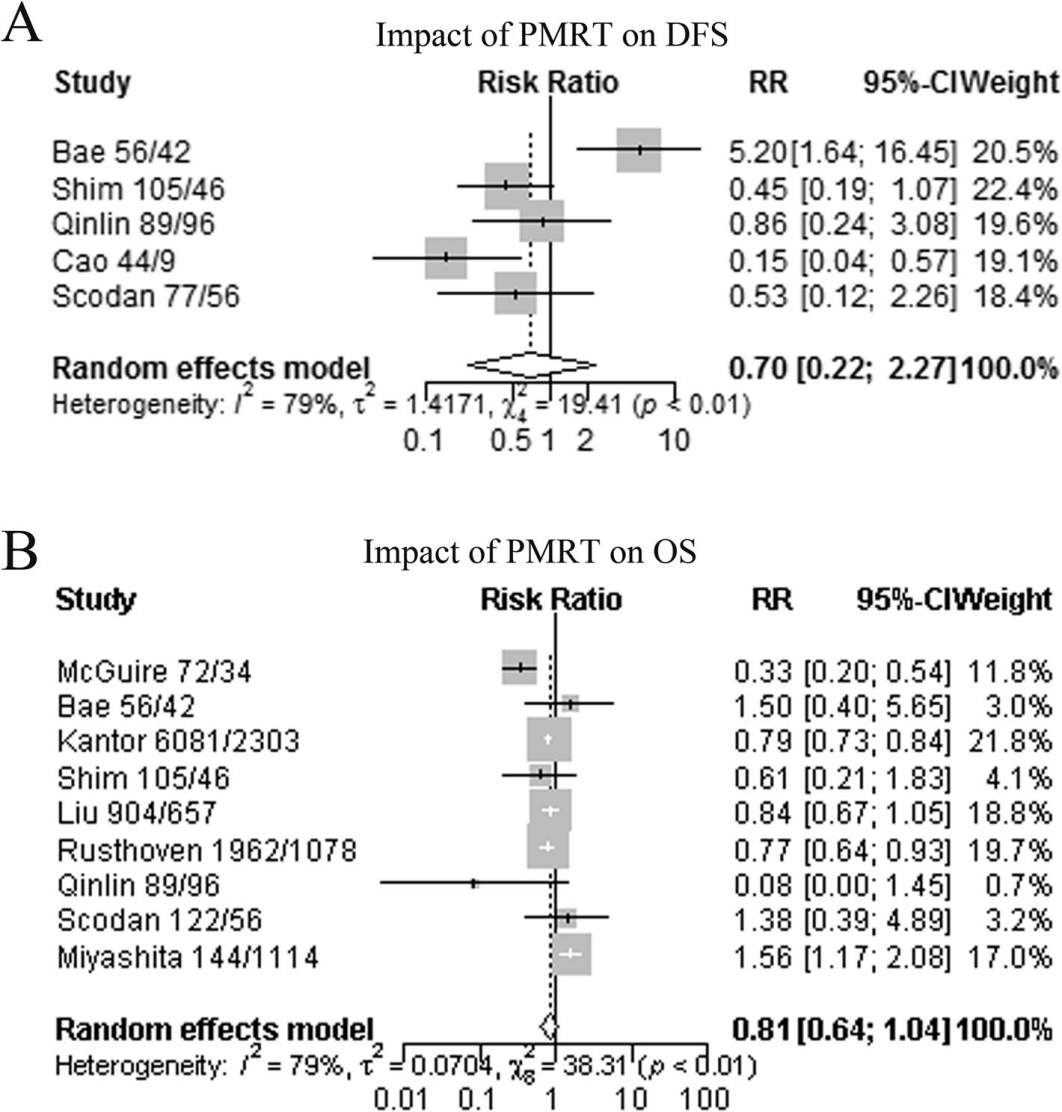
Meta-analysis of relative risk (RR) for the correlation between PMRT and survival outcomes. **A** DFS in PMRT and no-PMRT group; **B** OS in PMRT and no-PMRT group. RR less than 1 represents a lower risk of progression or death. The analysis showed that patients with PMRT were no correlation with a difference in DFS and OS. *Abbreviations*: PMRT, postmastectomy radiation therapy; DFS, disease-free survival; OS, overall survival

The pooled RR for DFS was available in five breast cancer studies. Since the heterogeneity among studies was significant (I^2^ = 79.0%, *P* < 0.001), the relationship between PMRT and DFS was evaluated using the random-effects model. The estimated pooled RR in the PMRT group was not associated with a high DFS rate compared with patients in the non-PMRT group (RR, 0.70; 95% CI, 0.21–2.27, *P* = 0.55; Fig. 3A). The results showed that no variable significantly influenced the RR estimate (Supplementary Figure S2 and Supplementary Table S1).

### Effects of PMRT on overall survival (OS) in patients with ypN0 breast cancer

PMRT impact on OS was available in nine ypN0 breast cancer studies. The pooled RR was used to analyze survival outcomes in patients who received PMRT. The random-models was applied, as heterogeneity across studies was significant (I^2^ = 79%, *P* < 0.001). The results showed no significantly longer OS (RR, 0.81; 95% CI, 0.64–1.04, *P* = 0.10, Fig. 3B) in patients in the PMRT group compared with those in the non-PMRT group. No significant publication bias was detected by the funnel plot and Egger’s test (Supplementary Figure S2 and Supplementary Table S1). In sensitive analysis, the results for OS showed the stability across the included studies.

## Discussion

Currently, there are no published randomized trials to guide the impact of PMRT on breast cancer patients treated with NAC, particularly in patients who are ypN0 and in whom the advantage of PMRT is not known. This meta-analysis is the first study to investigate the efficacy of PMRT in breast cancer patients who received NAC. We confirmed that radiation could reduce the LRR rate (*P* = 0.007), particularly in patients with stage III breast cancer (*P* < 0.001) after NAC and postmastectomy in ypN0 patients. In contrast, DFS (*P* = 0.55) and OS (*P* = 0.10) wasn’t significantly improved in the PMRT compared with the non-PMRT group. It was not surprising that the benefits of PMRT in the current study were similar to those reported after adjuvant chemotherapy [8, 22].

Multivariable analysis revealed that administration of PMRT to ypN2–3 women was associated with improved LRR, DFS and OS [23, 24]. Since node-positive is an independent prognostic factor in breast cancer, we wondered what indications had the potential to result in a down staging of the pathological extent of disease following NAC. A retrospective study revealed that in patients receiving primary systemic treatment and mastectomy, there was a significant reduction in the 5-year LRR rate in the PMRT group (24% vs. 4%, *P* < 0.001) after a median of 75 months of follow-up in 162 patients with ypN0 breast cancer [21]. In our analysis, the data also evaluated the benefit derived from PMRT in patients receiving NAC and mastectomy for their locally advanced breast cancer. The results identified PMRT as a significant predictor of reduced LRR (*P* = 0.007). These results are consistent with previous reports, including the studies from the MD Anderson Cancer Center.

Another major concern is whether PMRT improves survival outcomes in patients with ypN0 breast cancer after NAC. Recent retrospective data from Korea showed that PMRT was not associated with a difference in DFS or OS for patients with clinical stage II-III breast cancer who were ypN0 [15]. The present meta-analysis confirmed that PMRT did not improve DFS or OS in patients with ypN0 breast cancer who received PMRT following primary systemic treatment. Therefore, the preliminary insights from this analysis suggest that PMRT did not provide any survival benefit in patients, similar to what has been reported previously, although additional data are needed to confirm these findings. Nevertheless, a number of other risk factors, such as tumour stage, molecular typing of cancer and comorbidities, might affect both DFS and OS. The benefit of PMRT may be from a higher risk of persistent local-regional disease in ypN0 patients after NAC. It could reduce breast cancer mortality in patients with clinically lymph node-positive disease. Moreover, molecular typing of triple negativity is a significantly worse survival rate than patients with other subtypes. The omission of PMRT potentially places the patients at an increased risk of mortality. If the patients have a low competing risk of incurable persistent disease, it will also affect survival rate. Therefore, a longer follow-up time may allow the significant benefit seen in LRR to translate to an increased benefit in OS.

This study had some limitations. First, despite knowing the clinical disease stage before NAC, there may be other factors such as ER/PR status or pathologic stage after NAC that could influence patient selection for PMRT among those with ypN0 breast cancer. Second, this was a retrospective analysis where radiation was not a randomized variable. In an effort to reduce the potential for selection biases, this may have affected whether patients did or did not receive radiation. In addition, in subgroup analyses, for some of the subgroups, the sample size was limited, so we could not obtain conclusive results from patients with earlier stage disease.

## Conclusions

In summary, our results strongly suggest that PMRT is associated with reduced LRR, particularly in stage III breast cancer patients. However, PMRT was not associated with improved survival benefit among those with ypN0 breast cancer after NAC. A prospective randomized study, such as the ongoing NSABP B-51/RTOG 1304 trial (ClinicalTrials.gov, NCT01872975), will be needed to confirm our results and serve as guidance for selecting the optimal radiotherapy regimen for ypN0 breast cancer patients.

## Supplementary Information

**Additional file 1: Supplementary Figure S1.** The relationship between PMRT and LRR was evaluated using the random-effects model. (A) Stage I-II breast cancer; (B) Stage III breast cancer. *Abbreviations*: PMRT, postmastectomy radiation therapy; LRR, local-regional recurrence.

**Additional file 2: Supplementary Figure S2.** A funnel plot of studies that reported LRR or survival outcomes. (A) LRR; (B) DFS; (C) OS. *Abbreviations*: LRR, local-regional recurrence; DFS, disease-free survival; OS, overall survival.

**Additional file 3: Table S1.** Egger’s test of funnel plot asymmetry.

## Data Availability

All data generated or analysed during this study are included in this published article.
