# Supplementary material for: The role of postmastectomy radiation in patients with ypN0 breast cancer after neoadjuvant chemotherapy: a meta-analysis
Source: BMC Cancer. 2021 Jun 25;21:728. doi: 10.1186/s12885-021-08423-1 (PMC8234630; doi:10.1186/s12885-021-08423-1)
Supplement: Supplementary file 1 — Additional file 1: Supplementary Figure S1. The relationship between PMRT and LRR was evaluated using the random-effects model. (A) Stage I-II breast cancer; (B) Stage III breast cancer. Abbreviations: PMRT, postmastectomy radiation therapy; LRR, local-regional recurrence. [file 12885_2021_8423_MOESM1_ESM.docx]

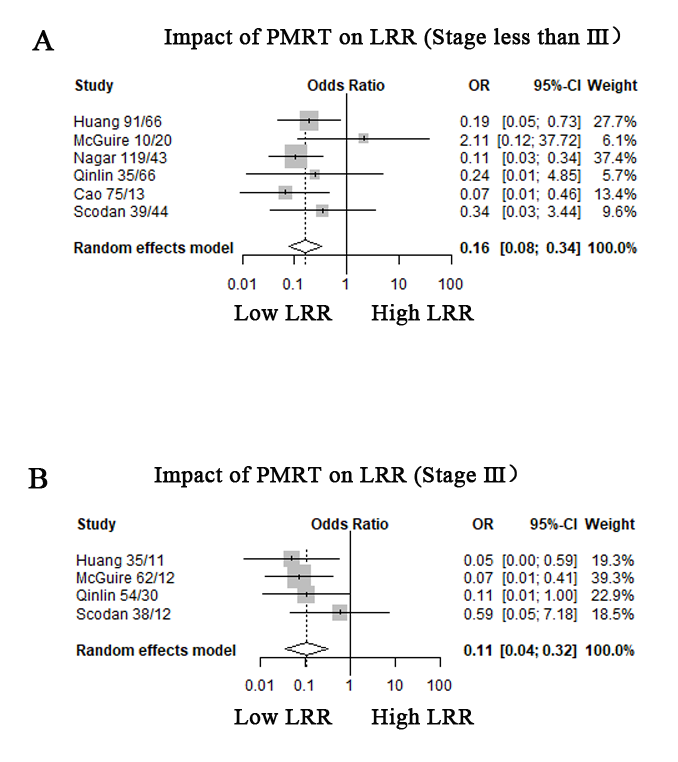


**Supplementary Figure S1.** The relationship between PMRT and LRR was evaluated using the random-effects model. (A) Stage Ⅰ-Ⅱ breast cancer; (B) Stage III breast cancer. *Abbreviations*: PMRT, postmastectomy radiation therapy; LRR, local-regional recurrence.
