# Supplementary material for: The role of postmastectomy radiation in patients with ypN0 breast cancer after neoadjuvant chemotherapy: a meta-analysis
Source: BMC Cancer. 2021 Jun 25;21:728. doi: 10.1186/s12885-021-08423-1 (PMC8234630; doi:10.1186/s12885-021-08423-1)
Supplement: Supplementary file 2 — Additional file 2: Supplementary Figure S2. A funnel plot of studies that reported LRR or survival outcomes. (A) LRR; (B) DFS; (C) OS. Abbreviations: LRR, local-regional recurrence; DFS, disease-free survival; OS, overall survival. [file 12885_2021_8423_MOESM2_ESM.docx]

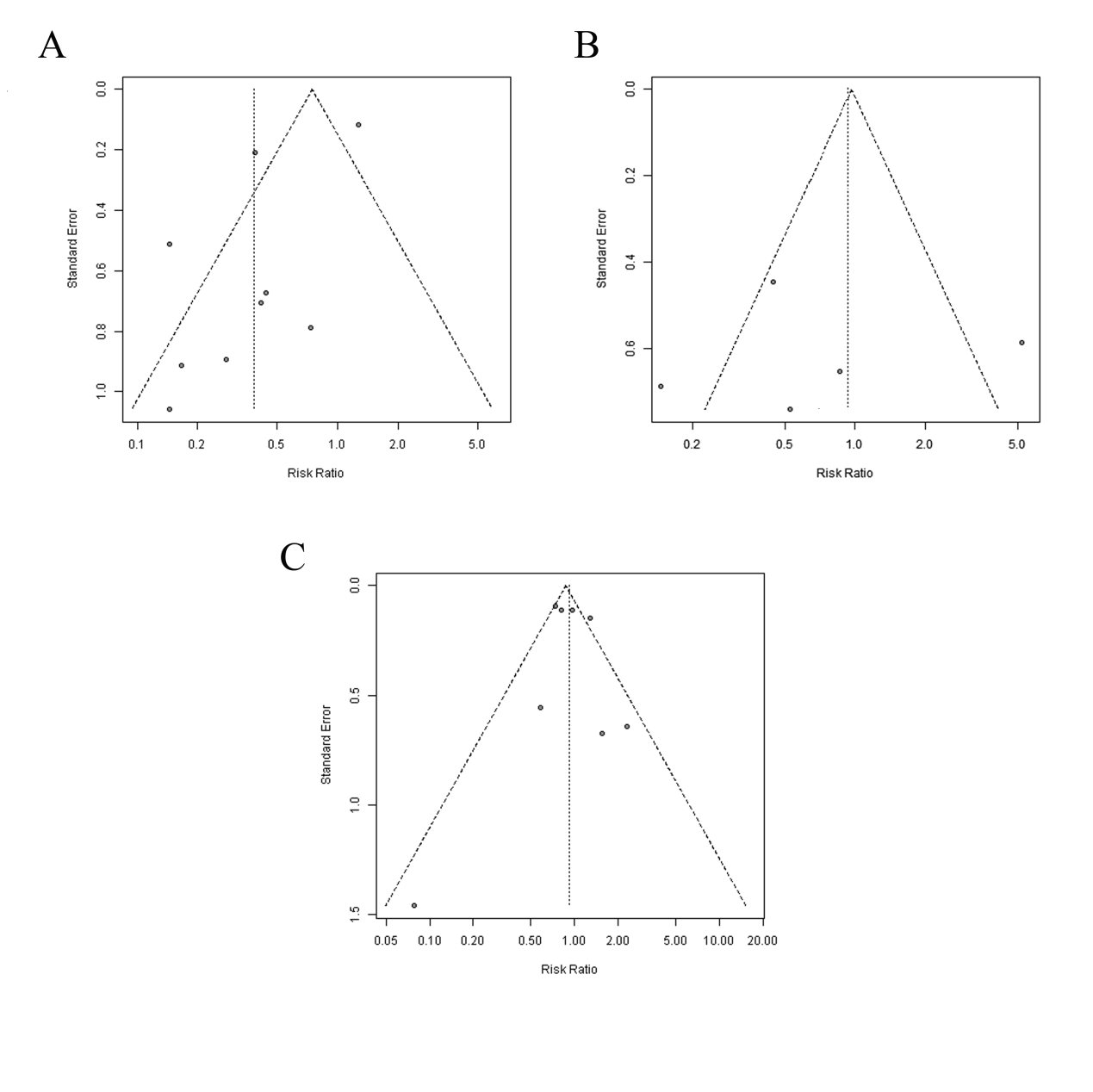
**Supplementary Figure S2.** A funnel plot of studies that reported LRR or survival outcomes. (A) LRR; (B) DFS; (C) OS. *Abbreviations*: LRR, local-regional recurrence; DFS, disease-free survival; OS, overall survival.
