# Supplementary material for: The role of postmastectomy radiation in patients with ypN0 breast cancer after neoadjuvant chemotherapy: a meta-analysis
Source: BMC Cancer. 2021 Jun 25;21:728. doi: 10.1186/s12885-021-08423-1 (PMC8234630; doi:10.1186/s12885-021-08423-1)
Supplement: Supplementary file 3 — Additional file 3: Table S1. Egger’s test of funnel plot asymmetry. [file 12885_2021_8423_MOESM3_ESM.docx]

Table S1 Egger's test of funnel plot asymmetry.

| Clinicopathological parameters | t value | df | *P* value |
| --- | --- | --- | --- |
| LRR (Overall population) | -2.58 | 7 | 0.037 |
| LRR (Stage < III) | 1.58 | 4 | 0.189 |
| LRR (Stage III-IV) | 0.40 | 2 | 0.725 |
| Disease free survival (DFS) | -0.06 | 3 | 0.954 |
| Overall survival (OS) | 0.01 | 7 | 0.990 |
